# Intravenous Treatment with a Long-Chain Omega-3 Lipid Emulsion Provides Neuroprotection in a Murine Model of Ischemic Stroke – A Pilot Study

**DOI:** 10.1371/journal.pone.0167329

**Published:** 2016-11-30

**Authors:** Dirk Berressem, Konrad Koch, Nicole Franke, Jochen Klein, Gunter P. Eckert

**Affiliations:** 1 Goethe-University of Frankfurt, Department of Pharmacology, Germany; 2 Justus-Liebig-University Giessen, Institute of Nutritional Sciences, Germany; Indian Institute of Integrative Medicine CSIR, INDIA

## Abstract

Single long-chain omega-3 fatty acids (e.g. docosahexaenoic acid (DHA) or eicosapentaenoic acid (EPA)) are known for their neuroprotective properties associated with ischemic stroke. This pilot study aimed to test the effectiveness of an acute treatment with a long-chain omega-3 lipid emulsion (Omegaven 10%^®^, OGV) that contains fish oil (DHA 18 mg/ml; EPA 21 mg/ml) and α-tocopherol (0.2 mg/ml) in a transient middle cerebral artery occlusion (MCAO) model of ischemic stroke in mice. For this purpose, female CD-1 mice were anesthetized and subjected to 90 minutes of MCAO. To reflect a clinically relevant situation for an acute treatment, either after induction of stroke or after reperfusion, a single dose of OGV was injected intravenously into the tail vein (5 ml/kg b.w.). A neurological severity score was used to assess motor function and neurological outcome. Stroke-related parameters were determined 24 hours after MCAO. Microdialysis was used to collect samples from extracellular space of the striatum. Mitochondrial function was determined in isolated mitochondria or dissociated brain cells. Inflammation markers were measured in brain homogenate. According to control experiments, neuroprotective effects could be attributed to the long-chain omega-3 content of the emulsion. Intravenous injection of OGV reduced size and severity of stroke, restored mitochondrial function, and prevented excitotoxic glutamate release. Increases of pro-inflammatory markers (COX-2 and IL-6) were attenuated. Neurological severity scoring and neurochemical data demonstrated that acute OGV treatment shortly after induction of stroke was most efficient and able to improve short-term neurological outcome, reflecting the importance of an acute treatment to improve the outcome. Summarising, acute treatment of stroke with a single intravenous dose of OGV provided strong neuroprotective effects and was most effective when given immediately after onset of ischemia. As OGV is an approved fishoil emulsion for parenteral nutrition in humans, our results may provide first translational data for a possible early management of ischemic stroke with administration of OGV to prevent further brain damage.

## Introduction

Ischemic stroke is a major cause of death worldwide and responsible for serious long-time disability in adults. Thrombolytic treatment provides benefits but only for a small subset of patients who are suitable for lysis therapy. Neuroprotective treatments are aimed at preserving neurons and preventing neurodegeneration but have not been proven effective in humans yet.[1] However, neuroprotection remains a prominent goal for stroke therapy and ischemia-related damage.[2] Ischemia induces changes in mitochondrial respiration and increased mitochondria-related oxidative stress.[3] Thus, mitochondria are an important target for neuroprotection in ischemic stroke.[4] Experimental studies identified intravenous administration of the long-chain omega-3 fatty acid docosahexaenoic acid (DHA)—a major component of fish oil—at least in the next 3 hours following initiation of stroke and 1 hour post-reperfusion as a potent neuroprotective agent in ischemic stroke.[5] It was concluded that DHA has the potential for treating focal ischemic stroke in a clinical setting and that acute administration of DHA enriched lipid emulsions may be an effective intervention in pathogenesis of human stroke.[6] Early discovery and prevention of long-term sequelae is the primary task in treating patients with acute ischemic stroke.[7] Therefore, we aimed to test the effectiveness of an intravenous injection (5 ml/kg b.w.) of OGV shortly after onset of ischemic stroke or after reperfusion in a transient MCAO mouse model. This situation should reflect two clinically relevant points in time or situations: First, an early neuroprotective treatment in patients arriving at the hospital with a suspected ischemic stroke (at stroke). Secondly, a neuroprotective treatment in patients with diagnosed ischemic stroke after lysis or removal of the thrombus (at reperfusion). The quantity of OGV used in this study corresponds to a human dose of 0.41 ml/kg.[8] This dose is in the lower range for human use because OGV is approved for doses up to 2 mL/kg body weight. OGV is an iso-osmolar lipid emulsion already in clinical use for parenteral nutrition and contains fish oil (DHA 18 mg/ml; EPA 21 mg/ml) and α-tocopherol (0.2 mg/ml). We decided to use OGV in a transient MCAO mouse model because our findings would be transferable to a clinical setting, giving a potentially translational outcome. In comparison, earlier studies used free DHA dissolved in saline which is less suitable for the intended human use.[9–11] Stroke related parameters were investigated 24 hours after reperfusion and showed reduced infarct size and infarct severity, improved neurological outcome and behavior, improved mitochondrial function, enhanced glucose levels, prevention of excitotoxic glutamate release and decreased neuroinflammation.

## Materials and Methods

### Animals and experimental stroke model

Female CD-1 mice (27-29g) were purchased from Charles River (Sulzbach, Germany) and kept under standardized conditions: 12 h light/dark cycle, temperature (22°C), humidity (70%) and access to food and water *ad libitum*. All animal procedures were carried out in order to minimize animal suffering according to German and European law. This study was registered and approved by local authorities (Regierungspräsidium Darmstadt, Antrag F8/21) and performed by experienced persons with adequate training conforming to the requirements of the Federation of European Laboratory Animal Science Associations and the European Communities Council Directive (Directive 2010/63/EU). Omegaven 10%^®^ (Lot. 16GH0062, containing 18.1 mg/mL DHA and 21.3 mg/mL EPA) was provided by Fresenius Kabi Deutschland GmbH, Bad Homburg, Germany.

Transient in vivo-ischemia was provoked by occluding the middle cerebral artery (MCAO) in the left hemisphere of mice. Mice were anesthetized with isoflurane (Forene^®^, AbbVie Deutschland GmbH & Co. KG, Germany) and maintained at 37°C and received 0.1 mg/kg buprenorphine as intraperitoneal injection (Temgesic^®^, Reckitt Benckiser Healthcare Ltd., UK) for analgesia. External carotid artery (ECA) and internal carotid artery (ICA) were dissected and a silicon coated suture (Doccol Corp., Redlands, Ca; USA) was introduced and pushed forward through the ICA until the coated top sealed the source of the middle cerebral artery (MCA). Proper positioning of the suture was verified by laser Doppler flowmetry.[12] Mice then recovered in their specified cage. After 90 minutes of occlusion, mice were re-anesthetized for suture withdrawal to allow reperfusion. For Sham group, surgery protocol ended after dissecting the ICA, thus no suture was inserted. In the acute post-operative phase (6 hours after surgery), animals were monitored hourly and every 6–8 hours subsequently. If signs of pain increased during this time, animals were eligible for additional analgesia of 0.1 mg/kg buprenorphine given intraperitoneally every 6–8 hours. 24 hours after reperfusion, animals were deeply anesthetized with isoflurane and euthanized by decapitation. In total, 146 mice underwent surgery, of which 28 died during the induction of the stroke and 7 during the recovery phase. No evidence of adverse effects was identified. The 35 animals who died during surgery or recovery phase were evenly distributed between the groups. Experiments have been repeated accordingly.

### Treatments

First experiments were carried out to prove potential neuroprotective effects of OGV related to its content of long-chain omega-3 fatty acids. A control medium-chain lipid emulsion Lipofundin MCT 10%^®^ (LPF, B Braun, Germany) and d-α-tocopheryl polyethylene glycol 1000 succinate (TPGS^®^, Sigma-Aldrich, Germany) were tested against saline and OGV in corresponding doses. LPF is also an approved lipid emulsion for parenteral nutrition but lacks long-chain omega-3 fatty acids whereas TPGS is a water-soluble derivative of α-tocopherol. A comparison of the average main ingredients of the two lipid emulsions OGV and LPF is summarized in Table 1.

**Table 1:** Comparison of average main-ingredients of Omegaven 10% and Lipofundin 10% MCT.

per 100 mL	Omegaven 10%	Lipofundin 10% MCT
Fishoil	10,00g	-
Soybean oil	-	5,00
Medium-chain-triglycerides (MCT)	-	5,00
Glycerol	2,50g	2,50g
α-Tocopherol	0,02g	0,01g
(3-sn-Phosphatidyl-)choline from egg	1,20g	0,8g

Throughout the experiments, OGV from a single batch was used to assure constant and comparable concentrations of its ingredients. Subsequently, mice were randomly divided and tested in two subprojects: In subproject 1 treatment took place 5 minutes after completion of the surgical procedures of transient MCAO (a.s., at stroke). In subproject 2 treatment took place after 90 minutes of transient MCAO (a.r., at reperfusion). Each subproject consisted of three groups: (1) Sham, (2) stroke control and (3) stroke OGV. Control and verum groups received a single bolus injection of approximately 150 μL (5 ml/kg b.w.) saline or OGV into the tail vein. According to earlier studies on long-chain omega-3 fatty acids and to exclude possible aggravating effects of other fatty acids such as n-6 fatty acids or saturated fatty acids, we chose saline as a control throughout the experiments.[9–11,13–16]

### Neurobehavioral Assessment

Behavioral tests were performed 1 hour before stroke surgery and 24 hours after reperfusion. Neurological impact was scored using a neurological severity score (NSS) adapted from published scores.[17,18] The NSS combines different measures of innate reflex, alertness, physiological behavior and motor ability to score neurological behavior. Neurological performance was scored on a scale of 0 to 16 (most healthy score = 0, most severe score = 16; refer to S1 Table).

### Infarct area and infarct severity

Infarct area was measured 24 hours after reperfusion. Brain slices were stained with a solution of 1% 2,3,5-triphenyl-tetrazolium chloride (TTC, Sigma-Aldrich, Germany) in phosphate buffered saline (PBS). Striatal brain slices from core region of ischemic stroke (due to MCAO) were used to determine the infarct areas and infarct severity as measured by differences in grayscales. Infarct volumes were determined by whole brain slices.

Stroke severity or amount of living tissue was determined by a grayscale analysis of TTC stained brain slices. The coloring agent allows to differentiate between metabolic active and inactive tissue in a continuous way, as density and intensity which is measured by grayscale varies depending on severity of stroke.[19] The colorless compound is transformed to a red dye by metabolic active tissue whilst metabolic inactive tissue remains white. The difference between the mean grayscale of impacted and healthy contralateral brain hemisphere was calculated for each animal. The lower the value, the more healthy tissue in the analyzed area, as a value of zero implies that there is no difference in the TTC stained color of impacted and contralateral brain hemisphere. Stroke area and grayscale analysis were achieved by Image J software (National Institutes of Health, USA. Version 1.46r).[20]

### Preparation of dissociated brain cells (DBC) for *ex vivo* studies

DBC were prepared according to a previously published method using separated hemispheres of brains.[21] Briefly, brain was washed and homogenized in a buffered medium, chopped and pressed through a nylon mesh for separation followed by several centrifugation steps. Subsequently on receipt of the pellet, DBCs were diluted in Dulbecco’s Modified Eagle Medium (DMEM) and seeded into 24 well plates or 96 well plates. Cells were cultured at 37°C under 5% CO_2_. DBCs were used for analysis of MMP and ATP levels 24 hours after reperfusion.

### Mitochondrial function

*For Mitochondrial isolation*, separated hemispheres of brains were homogenized as previously decribed.[22] Briefly, the sample passes through different centrifugal steps after homogenization to isolate mitochondria which were finally injected into a respirometer (Oxygraph-2k, Oroboros, Innsbruck, Austria).

*Mitochondrial respiratory function* was assessed by measuring consumption of oxygen in an Oxygraph-2k respirometer. Titrations of substrates and inhibitors induced different respiratory states. Mitochondrial respiration was normalized to protein content and citrate synthase (CS) activity., respectively. CS activity and protein content were assessed in isolated mitochondria.

*Mitochondrial Membrane Potential* (MMP) measurement was assessed in DBCs using the fluorescence colorant rhodamine-123 (R123) for analysis. DBCs were incubated for 15 min (37°C, 5% CO_2_) with 0.4 μM R123. DBC were centrifuged (3000 rpm, 5 min) and washed with HBSS buffer (supplemented with Mg^2+^, Ca^2+^, and HEPES; pH 7.4; 37°C). After adding fresh HBSS to the DBCs, MMP was obtained by measuring the fluorescence of the colorant at an excitation wavelength of 490 nm and at an emission wavelength of 535 nm on a Victor X3 2030 multilabel counter (Perkin Elmer, Rodgau-Jügesheim, Germany). The fluorescence was recorded in four sequential runs and normalized to corresponding protein concentrations.

*ATP levels* were determined in DBCs using a ViaLight^®^ Plus bioluminescence kit (Lonza, Walkersville, USA). Briefly, DBCs were incubated with lysis reagent followed by incubation with monitoring reagent for additional 5 min. Bioluminescence (linearly associated to ATP concentration) was obtained by a luminescence reader (Victor X3 2030 multilabel counter, Perkin Elmer, Rodgau-Jügesheim, Germany). Concentrations were recorded in two sequential runs and normalized to corresponding protein concentrations.

### Microdialysis

Microdialysis was performed as previously described.[23] Briefly, self-made dialysis probe was implanted into mouse striatum one day prior to MCAO. Anesthesia was induced by 2.4% isoflurane with synthetic air and fixed in a stereotaxic framework. The probe (exchange length 2.5mm) was implanted AP +0.5mm; L +2.2mm; DV -3.8mm from bregma and fixed at this position with dental cement (Ivoclar Vivadent AG, Schaan, Liechtenstein). Mice recovered from surgery in their specified cages for 24 hours. Microdialysis was initiated 30min before MCAO and continued until 30min after reperfusion. Fractions were collected separately (before, during and after occlusion). Perfusion liquid was artificial cerebrospinal fluid (aCSF: 147 mmol/LNaCl; 4 mmol/LKCl; 1.2 mmol/L CaCl_2_ and 1.2 mmol/L MgCl_2_), the perfusion rate was 2 μL/min. After completion of the microdialysis, animals were sacrificed by decapitation. Metabolites were determined using a CMA-600 microanalyzer (CMA Microdialysis, Stockholm, Sweden).

### Protein quantification and Western blot analysis

Protein levels were determined by using Pierce BCA Protein Assay Kit (Thermo Scientific, USA). Brain homogenate of separated hemispheres was used for Western blot experiments. Samples were incubated with primary antibodies [(Cox-2 (sc-1745), Santa Cruz Biotechnology, USA; IL-6 (PP012P2), IL10 (PP007P2), Acris Antibodies, USA] and corresponding secondary antibodies (#401515, #401353 and #401253, Calbiochem, Germany) conjugated to horseradish peroxidase and visualized by ECLplus^TM^ Reagent (Amersham Biosciences, USA). Glyceraldehyde-3-phosphate dehydrogenase (GAPDH) detected with antibody (MAB374, Chemicon, Germany) and Tubulin detected with antibody (ab6160, Abcam, GB) served as loading control. Band detection and evaluation was achieved with BioRad’s Quantity One software.

### Statistics

All data are presented as means ± SEM. Statistical analysis was performed by applying t-test and Mann-Whitney test for groups of two and One-Way ANOVA with Tukey post test for groups of more than three (Prism 5.03, GraphPad Software, USA). A *P* value of < 0.05 was considered statistically significant. If not declared differently, significances always refer to control values.

## Results

### Neuroprotection by intravenous infusion of OGV at reperfusion

**Fig 1:**
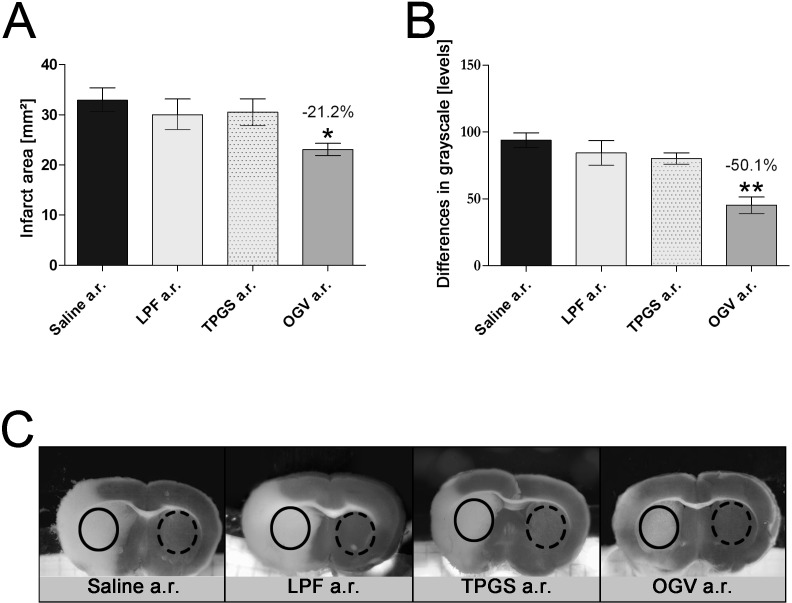
Effects of OGV and control emulsions when applied at reperfusion. Saline, Lipofundin® (LPF) and d-α-tocopheryl polyethylene glycol 1000 succinate (TPGS) in doses equal to OGV when injected at reperfusion (a.r.). (A) Infarct areas and (B) differences in grayscale for LPF, TPGS, and Omegaven 10% (OGV) vs. Saline; n = 6. Mean ± SEM, p*<0.05; p**<0.01; p***<0.001; One-Way ANOVA with Tukey post-test. (C) Representative striatal brain slices for determination of differences in grayscale for each group. Density of grayscale in a representative area from core of infarction (solid circle) was subtracted from corresponding grayscale of contralateral control area (dashed circle).

Transient MCAO for 90 minutes disrupted blood flow and caused ischemia in striatal and cortical areas of the brain as detected by laser Doppler (data not shown) and striatal glucose levels. LPF and TPGS, control items both lacking long-chain omega-3 fatty acids (refer to Table 1 for a list of main ingredients) neither affect stroke area (Fig 1A and 1C) nor stroke severity as measured by grayscale analysis (Fig 1B and 1C). OGV injection at reperfusion (a.r.) reduced the infarcted area by 21% and severity of stroke by 50% (Fig 1A, 1B and 1C).

**Fig 2:**
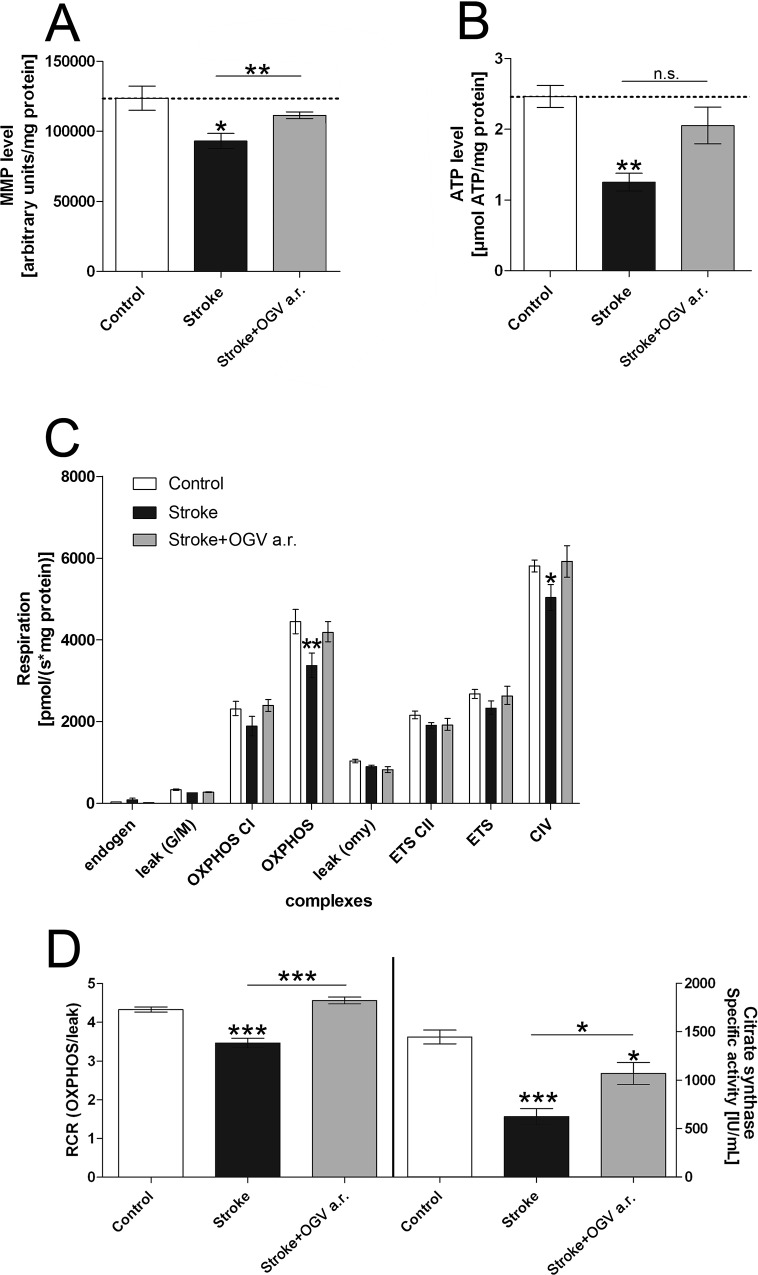
Marker of mitochondrial function after treatment at reperfusion. Sham-operated mice. (Control) versus stroke control group that received saline (Stroke) and stroke treatment group that received OGV at reperfusion (a.r.). (A) Mitochondrial membrane potential (MMP)- and (B) Adenosine triphosphate (ATP)-levels as measured 24 hours after reperfusion in dissociated brain cells, n = 8; (C) Respiration [pmol oxygen/(s* mg protein)] of different complexes of the respiratory chain were determined in isolated mitochondria, n = 6; (D) Respiratory control ratio (RCR) that indicates the coupling of mitochondrial respiration chain and citrate synthase activity that represents a quantitative marker for mitochondrial mass, n = 6. Mean ± SEM, p*<0.05; p**<0.01; p***<0.001; One-Way ANOVA with Tukey post-test.

Compared to the stroke control group, OGV a.r. increased the mitochondrial membrane potential by 20% (MMP; Fig 2A), and ATP levels by 63% (Fig 2B) which were significantly reduced in dissociated brain cells 24 hours after reperfusion. Protective effects of OGV on cerebral mitochondrial dysfunction were further investigated in isolated mitochondria.

Stroke significantly impaired the activity of OXPHOS and complex IV (cytochrome c oxidase) of the mitochondrial respiration chain (Fig 2C) and reduced the respiratory control ratio (RCR) and citrate synthase activity (Fig 2D). OGV improved the RCR by 31% (Fig 2D), indicating enhanced coupling of the respiratory system.[22,24] OGV also increased citrate synthase activity by 71% after stroke (Fig 2D), indicating enhanced mitochondrial mass.[25]

Although mitochondrial function as well as stroke area and severity of stroke were improved, OGV treatment at reperfusion resulted only in marginally improved neurological severity scores as early as 24 hours after reperfusion (data not shown).

### Superior effects of an acute OGV administration

**Fig 3:**
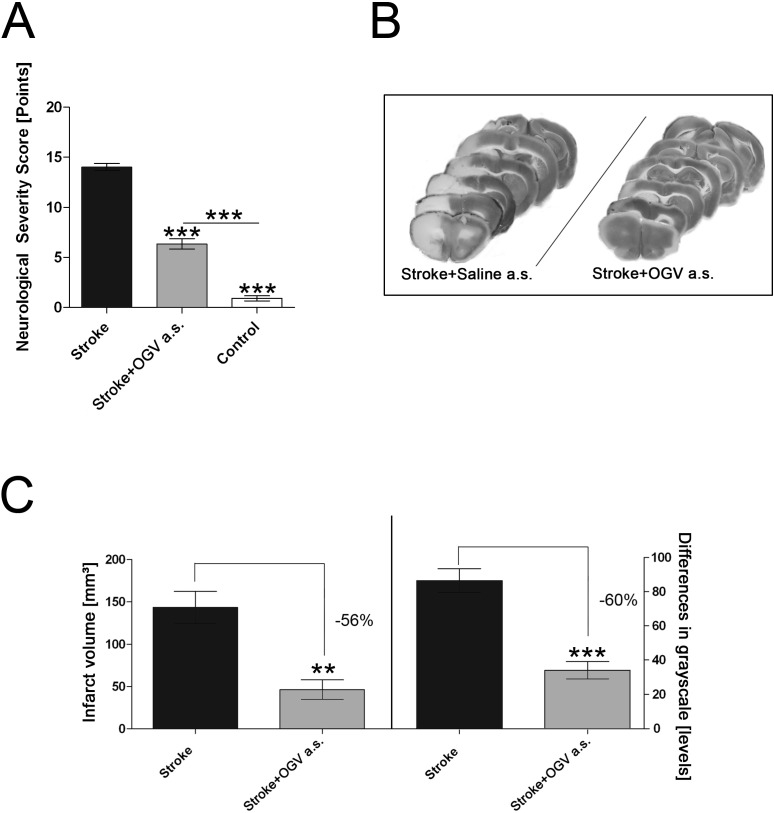
Effects of OGV when given at stroke (a.s.). (A) Neurobehavioral assessment (refer to S1 Table) for sham-operated mice (Control) versus control group that received saline (Stroke) and treatment group that received OGV at stroke (a.s.), (B) Representative brain slices for determination of infarct volume and differences in grayscale. (C) Effect on infarct volume and grayscale levels, n = 8. Mean ± SEM, p*<0.05; p**<0.01; p***<0.001; (A) One-Way ANOVA with Tukey post-test, (C) t-test.

Severe behavioral limitations (score 10 or 11) were manifest in all animals tested 24 h after 90 minutes of MCAO (Fig 3A). Significant improvement was observed when OGV was injected immediately after onset of MCAO (a.s.) (Fig 3A), resulting in a significant improved motor function.

**Fig 4:**
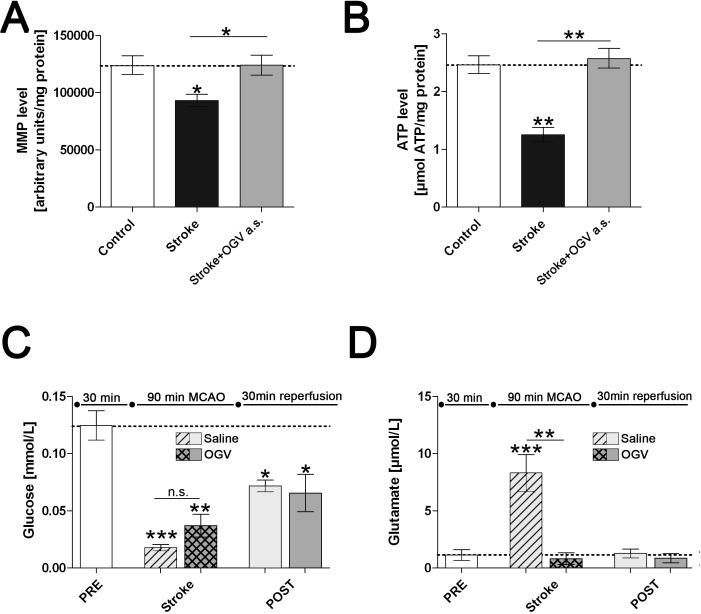
Marker of mitochondrial function after treatment at stroke. Sham mice (Control) versus stroke control group that received saline (Stroke) and stroke treatment group that received OGV at stroke (a.s.). (A) Mitochondrial membrane potential (MMP)- and (B) Adenosine triphosphate (ATP)-levels as measured in dissociated brain cells 24 hours after reperfusion. Energy metabolite levels in striatal core region of stroke for (C) glucose and (D) glutamate as determined by microdialysis 30 minutes before (PRE), 90 minutes during (Stroke) and 30 minutes after (POST) stroke surgery, n = 8. Mean ± SEM, p*<0.05; p**<0.01;p***<0.001; One-Way ANOVA with Tukey post-test.

MCAO led to steady necrotic lesions including both cortical and subcortical areas of the impacted, left hemisphere (Fig 3B). OGV injection a.s. reduced infarct volume by 56% as well as severity of stroke by 60% (Fig 3B and 3C) and restored mitochondrial function to basal values (Fig 4A and 4B).

Glucose and glutamate levels were determined before (PRE), during (Stroke) and after stroke (POST) in extracellular microdialysates collected in the striatum of the ischemic hemisphere (Fig 4C and 4D). Glucose levels dropped by 86% during stroke and increased after reperfusion (Fig 4C). Elevated glutamate levels by 740% of basal values were observed during MCAO (Fig 4D). OGV suppressed the release of glutamate during stroke (Fig 4D) and enhanced glucose levels in the impacted area (Fig 4C). Although, the OGV-induced rise in glucose levels was statistically not significant, the data indicate an improved blood supply into the affected tissue.

**Fig 5:**
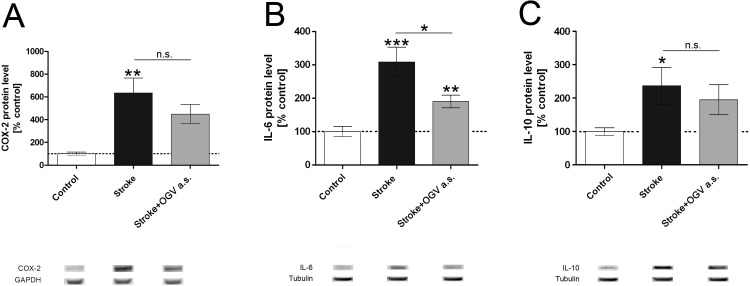
Western Blot analysis of brain homogenates. Sham-operated mice (Control) versus control group that received saline (Stroke) and treatment group that received OGV at stroke (a.s.). (A) COX-2-, (B) IL-6-, and (C) IL-10 protein levels, n = 8. Mean ± SEM, p*<0.05; p**<0.01; p***<0.001; One-Way ANOVA with Tukey post-test.

MCAO significantly increased pro-inflammatory protein levels of cyclooxygenase 2 (COX-2, Fig 5A) by 540% and interleukin-6 (IL-6 Fig 5B) by 220% as well as the anti-inflammatory marker Interleukin-10 (IL-10, Fig 5C) by 160%.

Compared to stroke, OGV a.s. significantly reduced the pro-inflammatory marker IL-6 by 49% (Fig 5B), but had no influence on IL-10 levels. All protein levels were measured 24h post-stroke.

## Discussion

The demand for long-chain omega-3 fatty acids escalates dramatically after ischemic stroke to limit the cellular damage by restoring destroyed neuronal membranes. During ischemia, polyunsaturated species from phospholipids (especially arachidonic acid and DHA) undergo significant hydrolysis which was not inversed through reperfusion.[26] We confirmed recent data demonstrating neuroprotective effects of DHA in experimental stroke models when applied at reperfusion.[5,11] Moreover, we report the novel finding that OGV injections shortly after onset of stroke provided superior neuroprotective actions (compared to treatment at reperfusion) on stroke volume, tissue health (as measured by grayscale analysis), mitochondrial function and development of inflammation in a murine MCAO model, emphasizing the importance of an earliest intervention to improve the outcome. OGV is an approved fish oil emulsion for parenteral nutrition containing long-chain omega-3 fatty acids (DHA 18 mg/ml; EPA 21 mg/,ml) and α-tocopherol (0.2 mg/ml). Thus, findings might be translated into a clinical setting. The dosing rationale for this study was based on recommendations of the GV-SOLAS (Gesellschaft für Versuchstierkunde–Society for Laboratory Animal Science) regarding the maximum feasible volume for intravenous substance application in the mouse, which is 5 ml/kg and is equal to our bolus injection of 150 μL in a mouse of approximately 30g weight.[27] This dose is 5 times below the accepted range of OGV in human. Belayev et al. reported toxic effects of 70 mg/kg DHA in rats. Doses of 3.5, 7, 14 and 35 mg/kg DHA showed equal beneficial effects.[11] Taking into account the conversion factor for doses from rats to mice, 3.5, 7, 14, 35 and 70 mg/kg in rats as reported by Belayev et al. correspond to doses of 7, 14, 28, 70 and 140 mg/kg in mice.[28] The bolus injection used in this pilot study contained 90 mg/kg DHA, which is below the dose that showed detrimental effects and within the beneficial dose that was reported by Belayev et al., thus reflecting a maximum non-toxic dose. This study was designed as a pilot study and should test a potentially translational treatment with long-chain omega-3 fatty acid emulsion approved for parenteral nutrition in humans. We identified two time points when treatment could take place in the best case: First, after induction of stroke but before diagnosis, and second, after diagnosis of stroke and lysis/removal of the thrombus. These points reflect the two earliest time points when treatment with OGV would be feasible and could potentially exert maximum effects. As patients with suspected or diagnosed ischemic stroke would receive a central venous access, we chose an intravenous injection as acute way of treatment. As stated above, we carefully chose one dose that should represent a maximum non-toxic dose based on earlier findings in the literature.[11] Beneficial effects of OGV can largely be assigned to their content of long-chain omega-3 fatty acids since experiments using comparable emulsions containing soy oil and medium-chain triglycerides (100 mg/ml) and α-tocopherol (0.2 mg/ml) (LPF or TPGS) in equivalent concentration showed no effects on stroke outcome. Beside DHA, OGV also contains the long-chain omega-3 fatty acid eicosapentaenoic acid (EPA) which also provides beneficial effects.[29] EPA also exerts anti-inflammatory as well as mild vasodilatory effects.[30,31] Exclusive treatment with EPA was shown to be ineffective in a model of hypoxic-ischemic brain injury, thus its neuroprotective potency seems to be lower than that of DHA which mediated strong neuroprotection in the same model.[6] EPA showed additional effects like vasodilatation and improved membrane fluidity that, in combination with DHA, may potentiate neuroprotective actions.[31] The biological precursor of long-chain omega-3 fatty acids DHA and EPA is the shorter-chain fatty acid alpha-linolenic acid (ALA). In the body, ALA is converted to DHA and EPA, but this conversion is ineffective. For example, conversion from ALA to DHA is only about 0–4%.[32] A recent study showed that acute intravenous injection of ALA was insufficient in improving motor recovery after ischemic stroke in mice, whilst dietary long-term supplementation with ALA was able to exert significant protective effects, emphasizing the importance and possibility of nutritional intervention in order to improve outcome in the critically post-stroke recovery phase.[33] Other studies claim that neuroprotective effects are widely attributed to conversion of ALA to DHA and that ALA seems not to be useful for an acute intervention of ischemic stroke.[34] Respective levels of ALA and EPA in the brain are 200–500 fold lower than of DHA, which might be attributed to a rapid metabolism via beta-oxidation and subsequently underline the brain’s need for DHA.[35] Otherwise, Bourourou et al. revealed that protective effects of ALA treatment are linked to an intrinsic effect rather than bioconversation of ALA to DHA as indicated by unchanged DHA levels of the brain after subchronic ALA injections.[33] As OGV also contains certain amounts of ALA, this might also contribute to the observed protective effects in addition to those mediated by e.g. DHA or EPA.[36] Additional data for ALA supplementation and its protective effects are also given by Blondeau et al. who summarizes that potential benefits of ALA are supported by animal studies as well as epidemiologic studies. He also supports the idea of an intravenous therapy targeting neurological conditions and speculates that this may offer significant benefits for patients suffering ischemic conditions.[37] The proposed intervention with OGV goes well with the supposed approach communicated by Blondeau et al. and supports this idea with first data.

In line with earlier reports, microdialysis showed significantly increased glutamate levels in the striatum of control mice which returned to basal levels at reperfusion.[38] Excessive glutamate release during ischemia leads to excitotoxicity that triggers apoptosis and finally neurodegeneration.[39,40] The excessive glutamate release peaks within minutes after ischemia and then slowly decreases within hours, emphasizing the importance of an early treatment if the glutamate mediated excitotoxicity should be targeted effectively to prevent further neuronal death.[12,23,41] We recently showed that the inhibition of the excessive release of glutamate triggered by ischemia results in a reduction of infarct area and enhances motor function.[20] Similarly, OGV treatment shortly after induction of MCAO abolished the release of extracellular glutamate levels and reduced the infarct area which might help to explain why only an acute treatment improved neurological outcome. Presumably, due to the fact that also treatment at reperfusion preserved significant amounts of viable brain tissue, long-term rehabilitation is expected to be better. Supplementation with fish oil was recently shown to be related to reduced cellular damage and enhanced cellular proliferation 6 weeks after ischemic stroke in the rat.[42] OGV numerically enhanced glucose levels. OGV enhances erythrocyte membrane fluidity and thus potently modulates endothelial cell integrity and exert vasodilatory effects which might explain an improved blood supply into the affected tissue, thus leading to slightly improved glucose levels obtained during microdialysis at stroke.[30,31,43] As a consequence, this mechanism might evoke concern about influences on bleeding times. Wachira et al. investigated the influence of (highly concentrated) long-chain omega-3 fatty acids on hemostasis and concluded that bleeding times did not exceed normal limits nor did produce clinically significant bleeding episodes and are rendered safe in monotherapy as well as combination therapy settings, although DHA and EPA exert a mild inhibition of platelet function that was often under detection limits. No support for discontinuing long-chain omega-3 fatty acid supplementation before invasive procedures was found. In contrast, their use improved clinical outcomes in several settings or reduced the risk of bleeding.[30] This finding is supported by a meta-analysis of clinical studies regarding relation of intake of long-chain omega-3 fatty acids and stroke by Larsson et al. who showed that no higher risks nor occurrences of hemorrhagic stroke could be stated, but a statistically significant reduction in stroke risk in relation to long-chain omega-3 fatty acid intake could be found for ischemic stroke that was only significant for ischemic but not hemorrhagic stroke. Larsson et al. attributed this statistical difference to the higher patient counts for ischemic stroke, resulting in higher statistical power.[44] A recent study focused on the concern of hemorrhagic transformation and tested whether DHA increases or decreases the risk of hemorrhagic transformation as a feared complication of cerebral ischemic infarction.[45] The results of this study showed that DHA attenuated hemorrhagic transformation and preserved integrity of the blood-brain-barrier whilst reducing inflammation.

Mitochondrial dysfunction is a major consequence of stroke that impacts brain tissue and causes elevated levels of reactive oxygen species (ROS) in the affected tissue.[46] Ischemia provokes negative alterations in mitochondrial respiration in animal models.[3] In mitochondria, efficacy of cellular respiration was significantly decreased. In contrast, the degree of lipid peroxidation mediated through increased formation of free radicals was significantly increased during ischemia and reperfusion.[20,26,47] In line with recent findings we report on impaired mitochondrial function after MCAO.[41] Intravenous treatment with OGV compensated for stroke-related decrease of mitochondrial respiration and significantly enhanced OXPHOS and cytochrome C oxidase activity. The OXPHOS system is the final biochemical pathway for the production of ATP. Thus, improved respiration led to an enhanced MMP that represents the propulsive power for ATP formation through F0/F1-ATPase (complex V) activity.[48] Accordingly, OGV significantly improved MMP and ATP levels as measured 24 hours after reperfusion.

Inflammation represents a severe and detrimental consequence of stroke that is characterized by an accumulation of inflammatory mediators.[49] Pro-inflammatory mediators such as COX-2 impair mitochondrial activity by inducing accumulation of distinct amounts of ROS through impairing OXPHOS activity.[50] Pro-inflammatory IL-6 is known to be elevated in the acute phase of stroke and high levels of IL-6 are associated with increased stroke volume and less favorable prognosis.[51] Its opponent IL-10 exerts anti-inflammatory effects, provides neuroprotection in ischemic stroke and reduced infarct volume at raised levels.[52] Levels of IL-6 (as part of the pro-inflammatory answer) and IL-10 (as part of the physiological repair mechanism to control the inflammatory answer) were significantly increased after transient MCAO which may involve inhibition of nuclear factor κB activation and an ERK mediated pro-survival cascade.[53] Several studies reported that treatment with anti-inflammatory drugs reduced infarct size and brain edema.[54] Long-chain omega-3 fatty acids are considered to be suppressors of neuroinflammation with several mechanisms of action.[35] Long-chain omega-3 fatty acids are actively biosynthesized to specialized pro-resolving mediators (SPM), a family of neuroprotective mediators that are involved in the resolution of inflammation.[55] This family is comprised of lipoxins, resolvins, protectins and maresins. Classic lipid mediators are formed from arachidonic acid and can exert pro-inflammatory (leukotrienes) or anti-inflammatory (lipoxins) actions. EPA can be biosynthesized to a class of resolvins (e-series resolvins) and DHA to several classes of resolvins (d-series resolvins), protectins and maresins which are all actively involved in the resolution of inflammation.[30,55–57] Resolution of inflammation by SPMs involves several mechanisms like neutrophil recruitment, stimulation of clearance mediated by macrophages, counterregulation of pro-inflammatory mediators such as COX-2 or IL-6 and tissue remodeling.[56] Neuroprotectin D1 (NPD-1) represents one of the first and most prominent neuroprotective metabolites of DHA which was identified by Bazan et al. earlier.[58,59] NPD-1 is a protectin that attenuates leukocyte infiltration, inhibits pro-inflammatory signaling and reduces infarct size in animal models of ischemic stroke via inhibition of Nf-κB pathway activation, increased expression of anti-apoptotic proteins like Bcl-2 and decreased expression of pro-apoptotic protein such as Bax.[5,60,61] Since DHA can be converted to several different classes of SPMs which each of them individually exert potent neuroprotective and anti-inflammatory effects, it is one of the most important members of long-chain omega-3 fatty acids.[56] OGV treatment significantly decreased protein levels of IL-6 confirming its anti-inflammatory properties.[62] Our experiments indicate that transient MCAO induced inflammatory and anti-inflammatory proteins and that OGV diminished the pro-inflammatory response only. A crucial point in time for inflammation is the moment of reperfusion. Paradoxically, reperfusion after removal of the thrombus does not improve the overall situation of the brain. As mitochondria experience dysfunction due to previously restricted supply with blood, glucose and oxygen, complexes of the respiratory chain do not work as intended: Components of the oxidative phosphorylation (OxPhos) are phosphorylized under physiological conditions and stabilize mitochondrial membrane potential in a range between 80–140 mV what is advantageous for the production of energy.[63] Following ischemia, several enzymes get activated that mediates dephosphorylation of components of the OxPhos which triggers a hyperactive state with a membrane potential above 150 mV. This hyperactivation does not result in consequences during ischemia due to the lack of oxygen. But after reperfusion, hyperactivated OxPhos components are supplied with oxygen, resulting in exponentially increased production of reactive oxygen species triggering further inflammation and apoptosis.[63] This detrimental phenomenon is called reperfusion-injury, emphasizing the need of an early intervention.[64]

Our data identified intravenous injection of OGV shortly after onset of transient MCAO as possible early medical management which could play a crucial role in inhibiting further brain damage mediated by ischemic stroke.[65] As literature did not reveal any concerns about possible influences of long-chain omega-3 fatty acids on bleeding times, treatment could also be started precautionary when stroke diagnosis is not fully performed yet. [30,44] Earliest neuroprotective treatment of ischemic stroke even before removal of thrombus showed best effects in our pilot study and was able to improve functional short-term outcome. This may be related to the reperfusion-injury that could be targeted by anti-inflammatory and anti-apoptotic long-chain omega-3 fatty acids and their neuroprotective metabolites when given during onset of ischemia. Although this pilot study showed short term effects only, it proved effectiveness of the proposed treatment. Further studies are needed to investigate the long-term effects of the proposed treatment and possibly further dosing regimens. Nevertheless, our results provide a translational approach that might contribute to a new and acute treatment of ischemic stroke in the clinics.

## Conclusion

In summary, treatment provided distinct neuroprotective properties in acute focal cerebral ischemia in mice by attenuating mitochondrial dysfunction as well as neuroinflammation and excitotoxicity. Neuroprotective effects were associated with the long-chain omega-3 fatty acid content of the lipid emulsion. Thus, intravenous administration of OGV might be an early and safe option for interventions even before diagnosis of ischemic stroke has been established. The results of this study might provide first translational data for an early clinical management of ischemic stroke with OGV which is pivotal to prevent further brain damage.

## Supporting Information

S1 Dataset Raw data of Fig 1. (PZF)

S2 Dataset Raw data of Fig 2. (PZF)

S3 Dataset Raw data of Fig 3. (PZF)

S4 Dataset Raw data of Fig 4. (PZF)

S5 Dataset Raw data of Fig 5. (PZF)

S1 Table List of tasks and scoring for the Neurological Severity Score (NSS). (PDF)
