# Supplementary material for: Intravenous Treatment with a Long-Chain Omega-3 Lipid Emulsion Provides Neuroprotection in a Murine Model of Ischemic Stroke – A Pilot Study
Source: PLoS One. 2016 Nov 30;11(11):e0167329. doi: 10.1371/journal.pone.0167329 (PMC5130273; doi:10.1371/journal.pone.0167329)
Supplement: S1 Table — (PDF) [file pone.0167329.s006.pdf]

Supplemental sheet

S1 Table. List of tasks and scoring for the Neurological Severity Score (NSS). Succession in the tasks will result in zero points, whilst task failure results in points of 1-3 depending on the task. The maximum score is 16. The higher the score, the worse the neurological outcome.

Task	Description	Success	Failure
**Exit circle**	Ability and initiative to exit a circle of 30 cm diameter within 3 minutes	0	1
**Mono-/Hemiparesis**	Paresis of upper and/or lower limb of contralateral side	0	1
**Straight walk**	Alertness, initiative and motor ability to walk straight once the mouse is put on the floor	0	1
**Startle reflex**	Innate reflex; the mouse will bounce in response to a loud clap of the hands	0	1
**Seeking behavior**	Physiological behavior as a sign of interest in the environment	0	1
**Beam balancing**	Ability to balance on a beam stick of 7 mm width for at least 10 seconds	0	1
**Round stick balancing**	Ability to balance on a round stick of 5 mm diameter for at least 10 seconds	0	1
**Beam walk: 3cm**	Ability to cross a 30cm long beam of 3 cm width	0	1
**Beam walk: 2cm**	Ability to cross a 30cm long beam of 2 cm width	0	1
**Beam walk: 1cm**	Ability to cross a 30cm long beam of 1 cm width	0	1
**Motor test**	Raising the mouse by the tail, flexion of fore- or hind limbs with a head movement <10° to vertical axis within 30 seconds	0	1
**Walking test**	Normal walk	0	
	Inability to walk straight	1	
	Circling towards the paretic side	2	
	Fall down to the paretic side	3	
**Pinna reflex**	Head shake when touching the auditory meatus	0	1
**Chimney test**	Ability to exit a chimney of 20cm length backwards in vertical coordination within 30 seconds	0	1
